# Two-Way Feature Extraction for Speech Emotion Recognition Using Deep Learning

**DOI:** 10.3390/s22062378

**Published:** 2022-03-19

**Authors:** Apeksha Aggarwal, Akshat Srivastava, Ajay Agarwal, Nidhi Chahal, Dilbag Singh, Abeer Ali Alnuaim, Aseel Alhadlaq, Heung-No Lee

**Affiliations:** 1 Department of Computer Science Engineering & Information Technology, Jaypee Institute of Information Technology, A 10, Sector 62, Noida 201307, India; apeksha.aggarwal@mail.jiit.ac.in or; 2 School of Computer Science Engineering and Technology, Bennett University, Plot Nos 8-11, TechZone 2, Greater Noida 201310, India; srivastavaakshat8@gmail.com; 3 Department of Information Technology, KIET Group of Institutions, Delhi-NCR, Meerut Road (NH-58), Ghaziabad 201206, India; ajay.agarwal@kiet.edu; 4 Nidhi Chahal, NIIT Limited, Gurugram 110019, India; nidhi.vce@gmail.com; 5 School of Electrical Engineering and Computer Science, Gwangju Institute of Science and Technology, Gwangju 61005, Korea; dilbagsingh@gist.ac.kr or; 6 Department of Computer Science and Engineering, College of Applied Studies and Community Services, King Saud University, P.O. Box 22459, Riyadh 11495, Saudi Arabia; abalnuaim@ksu.edu.sa (A.A.A.); asalhadlaq@ksu.edu.sa (A.A.)

**Keywords:** speech emotion recognition, machine learning, neural network

## Abstract

Recognizing human emotions by machines is a complex task. Deep learning models attempt to automate this process by rendering machines to exhibit learning capabilities. However, identifying human emotions from speech with good performance is still challenging. With the advent of deep learning algorithms, this problem has been addressed recently. However, most research work in the past focused on feature extraction as only one method for training. In this research, we have explored two different methods of extracting features to address effective speech emotion recognition. Initially, two-way feature extraction is proposed by utilizing super convergence to extract two sets of potential features from the speech data. For the first set of features, principal component analysis (PCA) is applied to obtain the first feature set. Thereafter, a deep neural network (DNN) with dense and dropout layers is implemented. In the second approach, mel-spectrogram images are extracted from audio files, and the 2D images are given as input to the pre-trained VGG-16 model. Extensive experiments and an in-depth comparative analysis over both the feature extraction methods with multiple algorithms and over two datasets are performed in this work. The RAVDESS dataset provided significantly better accuracy than using numeric features on a DNN.

## 1. Introduction

Speech is the most fundamental method of human communication. Humans can naturally detect the emotion in the speech they are presented with. However, it is not so straightforward for machines. This is where the importance of speech emotion recognition (SER) is highlighted. An SER system utilizes files containing speech data and classifies them into various emotions irrespective of the semantic contents [1]. A variety of emotions are generally classified by SER, including “happy”, “sad”, “angry” and “neutral”. SER systems aim to give rise to efficient methods of detecting emotions; this function is crucial for adding human elements like emotional response in machines. A well-working SER system can be utilized in several fields involving human-machine communication, ranging from mobile phone use to driving cars and beyond. Technologies like SER systems are getting increasingly important to reduce human time and effort. SER systems employ machines or robots to have a meaningful dialogue between a human and a machine. To create such a system, machine learning and deep learning models can be employed. This involves extracting significant features from raw data and utilizing them to make machines understand human emotions via model training [2]. During the training process, the model learns to classify information and produce desired outputs while maintaining a certain level of accuracy. Given the sheer number of options we can work with while crafting such a model, we have a huge number of permutations to try.

The task of emotion recognition from speech is divided into two major sections: feature selection and extraction, and classification. The authors in [3] have utilized a total of five features: Mel-Frequency Cepstral Coefficients (MFCC), Mel, Chroma, Tonnetz, and various permutations were tried and tested. The authors in [4] made use of a Decision Tree Classifier and a Convolutional Neural Network (CNN) to approach this problem. Authors extracted melcepst coefficients, randomize the data and then proceeded to train and test them over both the Decision Tree Classifier and the CNN. The highest accuracy that was achieved in this work was 72%. Similarly, the authors in [5] used the Random Forest Classifier and the Decision Tree Classifier to tackle the problem at hand. The best average recognition rate they reached was 66.28% by utilizing decision trees. Recently, authors in [6] also used Mel-Frequency Cepstrum Coefficients for recognizing emotions using speech, which have proven to be a crucial feature set for audio data.

Another effective and popular way to extract emotions from speech was to use CNNs [7,8] and Deep CNNs [9,10]. The authors in [11] used the Deep CNNs to approach the task. They managed the best test accuracy of 40.2% using two convolutional layers and two pooling layers to train and test the data. Support Vector Machines remained a popular choice in classification problems and the authors in [12] had done a comprehensive study utilizing the same. They used a biased SVM to approach the problem at hand. They achieved a maximum average accuracy of 58.24%.

Apart from CNNs [13,14,15], Long Short-term Memory Networks (LSTM) [16], DNN [17,18] and CNN-LSTM hybrids have also shown promising results in the field of emotion recognition. LSTMs are more advanced Recurrent Neural Networks (RNN) optimized to use gates to control information flow. Pandey et al. [19] adopted this methodology for the experiments. They extracted magnitude spectrograms, log-mel spectrograms, and MFCC and used a CNN-LSTM hybrid to train and test the data. The authors achieved an accuracy of 82.35% by using MFCCs as their input. However, only four emotions were tested in this work.

The authors in [20] utilized deep neural networks to calculate the weighted and unweighted accuracy of the model. This approach yielded a maximum weighted accuracy of 70.1% and maximum unweighted accuracy of 60.7%. Attention pooling is another option to tackle SER. The authors in [21] utilized attention pooling-based CNN and managed a weighted accuracy of 71.75% and an unweighted accuracy of 68.06%. Palo et al. [22] proposed another technique in which authors extracted different features and calculated the maximum accuracy achieved. The authors in this work achieved the best performance by using MFCCs when only four emotions (bored, angry, sad, and surprised) were taken into account. A similar study was done in [23] where authors utilized Log Mel-Spectrograms, MFCCs, eGeMAPS, and Prosody. Log Mel-Spectrograms gave the best accuracy when using a CNN (55.92%) and MFCCs showed the best accuracy when using an Attentive CNN (56.10%). In another work [24], different ANN models were used to calculate the accuracy of emotion prediction. The authors achieved the best results by using a frame-based CNN network, which gave a test accuracy of 64.78%. Lech et al. [1] proposed SER system which utilized AlexNet; a pre-trained image classification network and achieved the average accuracy of approximately 80%.

Another popular approach to the problem is making use of RNNs of various types, one such approach was proposed in [25], where authors utilized a CNN-RNN hybrid (CRNN) to address speech emotion recognition. They achieved a maximum unweighted accuracy of 63.98% by utilizing an HSF-CRNN system. In another experiment involving an RNN, the authors at [26] used an LSTM Neural Network to achieve an unweighted accuracy of 60.02%. The authors in [27,28,29] used similar RNN and LSTM systems to approach SER, achieving accuracies of 63.5%, 58.7%, and 63.89%, respectively.

For the present work, we have worked upon one-dimensional and two-dimensional data. For one dimensional data, we have used three different classifiers: Decision Tree [30], Random Forest [31], and MLP [32]. For two-dimensional data, although the primary focus has been on deep neural networks, we have also proposed the use of a dummy classifier to set a baseline accuracy. We have generated mel spectrograms [33] as 2D features for this research in addition to the generation of 1-D features from the audio dataset to test the model on four specific emotions: happy, sad, angry, and neutral. The contribution of this paper are summarized as follows:

1. Two-way feature extraction is proposed by utilizing super convergence to extract two sets of potential features from the speech data.
2. Principal component analysis (PCA) and deep neural network (DNN) with dense and dropout layers are applied to the features obtained from the proposed two-way feature extraction model.
3. The pre-trained VGG-16 model is also trained on the features from the proposed two-way feature extraction model.
4. Multimodal speech data is utilized for training.

## 2. Materials and Methods

**Figure 1:**
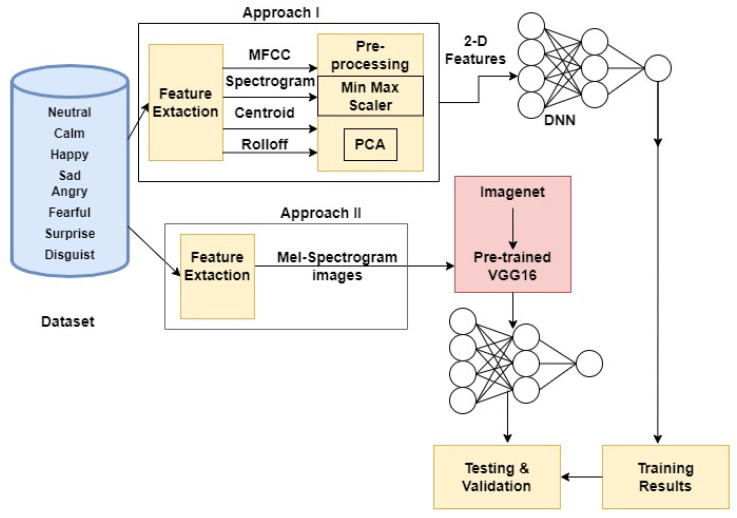
Schematic representation of two-way feature extraction for speech data.

In this work, we have proposed a two-way approach to extract features from the speech dataset. Schematic representations of the steps are depicted in Figure 1. Both the approaches are described further in this section.

### 2.1. Dataset Description

For this research work, two datasets have been utilized. First is the Toronto Emotional Speech Set (TESS) [34] and the second one is the Ryerson Audio-Visual Database of Emotional Speech and Song (RAVDESS) [35]. TESS consists of a set of 200 target words; every target word is spoken post the phrase “Say the word". The same has been recorded by two actresses portraying seven emotions (anger, disgust, fear, happiness, pleasant surprise, sadness, and neutral). There are a total of 2800 data samples in the dataset. RAVDESS consist of 24 actors, 60 trials per actor. A total of eight emotions are portrayed (calm, happy, sad, angry, fearful, surprise, and disgust) in this dataset. There are a total of 1440 data samples in the dataset.

### 2.2. Approach I

In the present work we have proposed two approaches to extract two types of features. In the first approach we work directly on the audio dataset to obtain numerical features. This section describes the details further.

#### 2.2.1. Feature Extraction

To anticipate the emotion of a given speech, we need to identify and extract one or more meaningful features. An audio dataset is rendered suitable by extracting suitable features from voice signals. In this approach, a combination of MFCC, Log Mel-Spectrogram, Chroma, Spectral centroid, and Spectral rolloff have been extracted. The librosa library has been used for feature extraction from audio signals directly. Post the extraction, the features of each file along with the labels have been converted to a 2-D feature vector.

#### 2.2.2. Dimensionality Reduction and Preprocessing

A total of 180 features have been extracted from the audio files. To remove the sparsity and high dimensionality of the dataset, further pre-processing of the data have been performed. Firstly, the data have been normalized using the MinMaxScaler from sklearn. Furthermore, to reduce the dimensionality, PCA has been utilized. PCA is used as it reduced the overfitting significantly by eliminating highly correlated variables. Using PCA, 80 important features have been selected to allow for effective training and testing.

#### 2.2.3. Model Architecture

**Figure 2:**
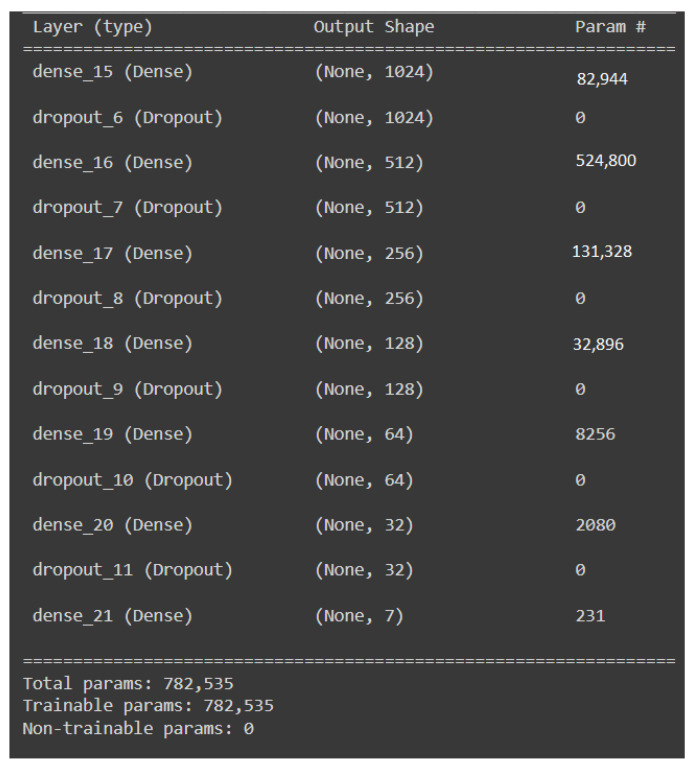
Architecture of the DNN Model.

In the first approach, DNN [36,37] is used along with dropout layers. The architecture for the DNN is shown in Figure 2. We have used a 13 layers for the network architecture viz. Seven dense layers and six dropout layers. For the dense layers, 1024 units have been used in the input layer and 512, 256, 128, 64, and 32 units have been used for the rest of the hidden layers, respectively. Since there are eight classes taken into consideration, the output layer consists of eight units. For the input and hidden layers, ‘relu’ has been used as the activation function, and for the output layer, the ‘softmax’ activation function [37,38] is utilized. Each of the input and hidden layers is followed by a dropout layer with a rate of 0.2. The model has been trained for a total of 400 epochs, with ‘rmsprop’ as the optimizer and ‘categorical crossentropy’ as the loss function.

### 2.3. Approach II

In this section, the second approach for feature extraction is described. In this approach, we have utilized spectrograms as image features.

#### 2.3.1. Feature Extraction

In this approach, we have used another method of feature extraction by employing transfer learning. In this approach, we have generated Log Mel-Spectrogram images from the input audio dataset. Using the librosa library, the Mel-Spectrogram image for each file has been extracted and saved, respectively, to the particular emotion class. In summary, a total of 1440 and 2800 images have been extracted for RAVDESS and TESS datasets, respectively.

#### 2.3.2. Model Architecture

We have further utilized VGG16’s *fastai* implementation. VGG16 is a Convolution Neural Network-based transfer learning model. It makes use of Conv2d layers along with BatchNorm2d and MaxPool2d layers. There are a total of 16 layers in VGG16.

### 2.4. Experiments

For the first approach, the data have been split into training and testing data with a train_size of 0.8 and test_size of 0.2. The extracted numeric features have been passed on to this DNN for training. Dropout layers have been used to prevent any overfitting. Furthermore, the target labels have been label encoded into categorical values. For constructing the model, we have utilized the Keras library. After every Dense layer, a dropout layer has been added. Softmax has been used as the activation function and categorical cross-entropy has been used as the loss function. The data have been trained for a total of 25 epochs for the TESS dataset and 400 epochs for the RAVDESS dataset, respectively.

For the second approach, the model was loaded with imagenet weights and the data have been trained for a total of five epochs for the TESS dataset and 25 epochs for the RAVDESS dataset.

## 3. Results

This section describes the results obtained from both the approaches over SER. Multiple evaluation metrics such as accuracy score, loss, classification report, and confusion matrix have been utilized throughout this research for evaluation. This section presents a detailed analysis of the performance of the previously described models on both datasets over multiple evaluation metrics.

### 3.1. Results on Approach I

**Figure 3:**
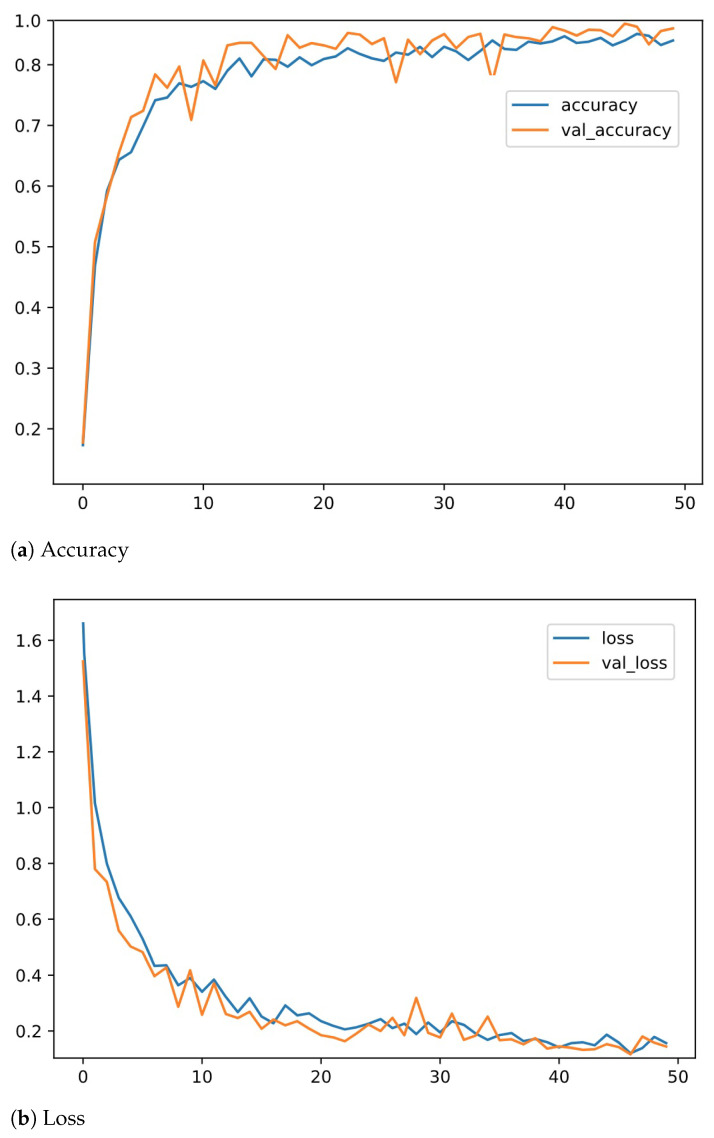
Accuracy and loss analyses of the proposed two-way feature extraction-based DNN on TESS dataset.

**Figure 4:**
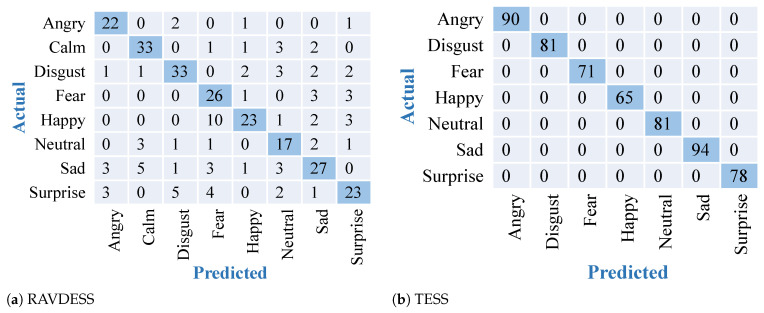
Confusion matrices for feature extraction and modeling using DNN over 2 datasets. Diagonal elements with dark blue color show accurately predicted classes.

The results obtained after training and testing the TESS dataset on the described DNN model are shown in Figure 3. Similar results have been found on the other datasets. The confusion matrices obtained after training and testing using the described DNN model on both the datasets are shown in Figure 4. Results depict high accuracy on both datasets. The confusion matrix shows more than 90% of samples are correctly classified for all the emotions. Confusion matrices in Figure 4 give a representation of the accuracies of the eight classes present in the RAVDESS dataset and seven classes present in the TESS dataset over DNN. Results show the outperformance over the TESS dataset with extremely low misclassifications.

### 3.2. Results on Approach II

**Figure 5:**
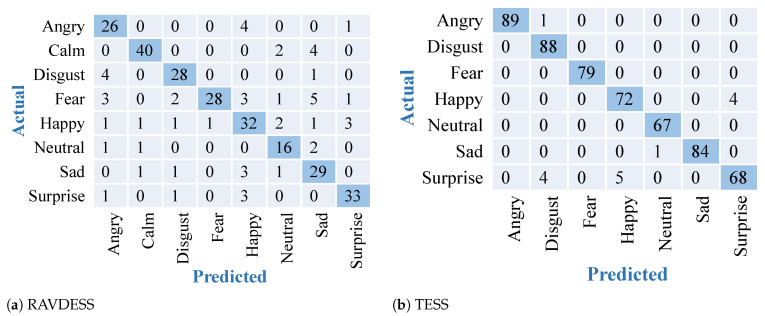
Confusion matrices for feature extraction and modeling using VGG16 over 2 datasets. Diagonal elements with dark blue color show accurately predicted classes.

The results obtained after training and testing of the RAVDESS dataset and the TESS dataset on the described VGG 16 transfer learning model are shown in Figure 5. Confusion matrices in Figure 5 represent the accuracies of the eight classes present in the RAVDESS dataset and seven classes present in the TESS dataset, obtained using VGG-16. This model gave us better accuracy than the DNN with low misclassifications. The results obtained are better than the previous approach. Due to the inbuilt feature extraction capability of VGG16 this model was able to outperform the other approach by giving an accuracy of 81.94% on the RAVDESS dataset and accuracy of 97.15% on TESS dataset.

### 3.3. Comparison with State-of-Art Approaches

In this section, we have compared the results of the proposed approach with the existing state-of-the-art. Table 1 shows a comparative study with respect to research groups addressing speech emotion recognition using deep learning models.

### 3.4. Comparative Analysis

**Figure 6:**
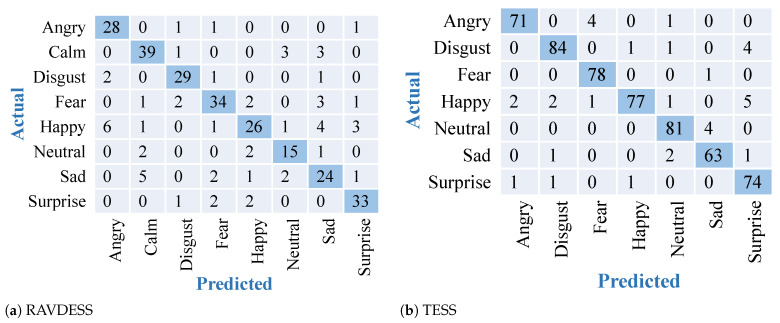
Confusion matrices for feature extraction and modeling using Resnet-18 over 2 datasets. Diagonal elements with dark blue color show accurately predicted classes.

To validate the effectiveness of the proposed approach, in addition to comparing results for two datasets, we have also compared results with the existing deep learning model of ResNet18. ResNet18 is an extremely popular CNN-based transfer learning model consisting of 18 layers. It has been loaded with the same weights and settings as VGG16. The results obtained after training and testing the RAVDESS dataset on the two datasets are shown in Figure 6. It shows the confusion matrices representing the best-performing models for the RAVDESS dataset. Out of the three models depicted in Figure 4, Figure 5 and Figure 6, VGG-16 outperformed with highest accuracy and best classification over the RAVDESS dataset. Table 2 shows the accuracy scores for all the models over both the datasets. It depicts the highest and most stable accuracies obtained by the proposed approach utilizing the VGG16 model and 2-D feature extraction. ResNet-18 also showed comparatively high accuracy over TESS dataset. This result variation in the second case could be because in some cases ResNet-18 handles gradients differently than VGG-16.

### 3.5. Comparison with Benchmark Algorithms

We have further compared the proposed approach with multiple deep learning and machine learning state-of-the-art models. For comparison, we have utilized four benchmark models of Decision Tree Classifier, Random Forest Classifier, MLP Classifier, and ResNet18. All these classifiers have been utilized and set to their base configuration from sklearn. The model configuration of ResNet18 was set the same as VGG16 to keep effective comparison. The comparison of accuracy scores of the benchmark models and proposed models is shown in Table 3. It suggests the highest accuracies obtained by the proposed approaches with respect to other SOTA models.

## 4. Conclusions

Identifying and processing human emotions via words is a challenging task. With the advent of machine learning and deep learning, several researchers have tried to address this. In the present work, a speech emotion recognition model has been proposed by using two-way feature extraction and deep transfer learning. Initially, two-way feature extraction has been proposed by utilizing the superconvergence to extract two sets of potential features from the speech data. Further, PCA is applied to the obtained first feature set. Thereafter, DNN with dense and dropout layers have been implemented on the important features obtained using PCA. On the other hand, a pre-trained VGG-16 model is applied to the second set of features to build the second model. Extensive experiments have been drawn and comparative analyses is performed in this work. Results revealed that the proposed models outperform the existing models in terms of various performance metrics. There are several limitations of this work, which can be the extension of this work in the future. The RAVDESS dataset consists only of North American speakers. Hence, the proposed approaches might give significantly less accuracy for people from different geographical areas. In future, we would like to apply the proposed model and other datasets as well. Similarly, this dataset takes into consideration people of median age. In future, we would like to extend this study to the vast vicinity characteristics of the subjects.

## Figures and Tables

**Table 1:** Comparative study.

SERIAL NO.	APPROACH	MODEL USED	DATASET USED	ACCURACY
01	Dissanayake [39]	CNN-LSTM (encoder)	RAVDESS	56.71%
02	Li et al. [40]	Multimodal Fine-Grained Learning	RAVDESS	74.7
03	Xu et al. [41]	Attention Networks	RAVDESS	77.4%
04	Proposed II Approach	2-D Feature Extration + VGG-16	RAVDESS	81.94

**Table 2:** Comparative analysis of RAVDESS and TESS.

SERIAL NO.	MODEL	RAVDESS	TESS
01	ResNet18	79.16%	96.26%
02	Proposed-I	73.95%	99.99%
03	Proposed-II	81.94%	97.15%

**Table 3:** Comparative analysis of RAVDESS and TESS.

SERIAL NO.	MODEL	RAVDESS	TESS
01	Decision Tree	37.85%	3.21%
02	Random Forest	46.88%	7.68%
03	MLPClassifier	33.68%	15.54%
04	ResNet18	79.16%	96.26%
05	Proposed-I	73.95%	99.99%
06	Proposed-II	81.94%	97.15%

## Data Availability

Not applicable.
